# Integrated Transcriptome and 16S rDNA Analyses Reveal That Transport Stress Induces Oxidative Stress and Immune and Metabolic Disorders in the Intestine of Hybrid Yellow Catfish (*Tachysurus fulvidraco*♀ *× Pseudobagrus vachellii*♂)

**DOI:** 10.3390/antiox11091737

**Published:** 2022-08-31

**Authors:** Tao Zheng, Yifan Tao, Siqi Lu, Jun Qiang, Pao Xu

**Affiliations:** 1 Wuxi Fisheries College, Nanjing Agricultural University, Wuxi 214081, China; 2 Key Laboratory of Freshwater Fisheries and Germplasm Resources Utilization, Ministry of Agriculture and Rural Affairs, Freshwater Fisheries Research Center, Chinese Academy of Fishery Sciences, Wuxi 214081, China

**Keywords:** transport stress, intestine, transcriptome, 16S rDNA, hybrid yellow catfish

## Abstract

Live fish are often transported in aquaculture. To explore the effects of transport stress, hybrid yellow catfish (*Tachysurus fulvidraco*♀ × *Pseudobagrus vachellii*♂) were subjected to simulated transport treatments (0–16 h) with 96 h of recovery after the 16-h transport treatment, and intestinal biochemical parameters, the transcriptome, and gut microbiota were analyzed. Transportation affected the number of mucus cells and led to oxidative stress in the intestine, which activated immune responses. Changes in lipid metabolism reflected metabolic adaptation to oxidative stress. Toll-like receptor signaling, peroxisome proliferator-activated receptor signaling, and steroid biosynthesis pathways were involved in the transport stress response. Gene expression analyses indicated that transport-induced local immune damage was reversible, whereas disordered metabolism recovered more slowly. A 16S rDNA analysis revealed that transport stress decreased the alpha diversity of the gut microbiota and disrupted its homeostasis. The dominant phyla (Fusobacteria, Bacteroidetes) and genera (*Cetobacterium*, *Barnesiellaceae*) were involved in the antioxidant, immune, and metabolic responses of the host to transportation stress. Correlation analyses suggested that gut microbes participate in the transport stress response and the host–microbiota interaction may trigger multiple events in antioxidant, immune, and metabolic pathways. Our results will be useful for optimizing transport processes.

## 1. Introduction

Transport is a necessary and routine operation in aquaculture management under intensive farming conditions [1]. Multiple stressors during transport, such as turbulence, congestion, and the deterioration of water quality can cause oxidative stress, dysfunctions of immunity and metabolism, and even the death of fish [2,3]. The skin, gills, and intestinal tract constitute the three major mucosal systems of fish [4]. Previously, we reported that transportation affects the antioxidant and immune systems of the skin and gills of hybrid yellow catfish (*Tachysurus fulvidraco*♀ × *Pseudobagrus vachellii*♂) [5,6]. This prompted us to determine the effects of transport stress on the intestinal tract to better understand the negative effects of transport on the mucosal system of fish. 

Several studies have focused on the response to transportation stress and its underlying mechanism in aquatic animals, using simulated transportation treatments applied under laboratory conditions [7,8]. For example, 4 h of transportation at a density of 200 g/L was found to induce oxidative stress, immunosuppression, and hydromineral imbalances in common carp (*Cyprinus carpio*) [9]. Thirty minutes of simulated transport significantly affected the liver function parameters, serum antioxidant status, and immune parameters of juvenile puffer fish (*Takifugu rubripes*) [10]. Compared with non-transported channel catfish (*Ictalurus punctatus*), those subjected to a 24-h transportation treatment had significantly lower fat, energy, and water contents in muscle [11]. Transportation often causes tissue damage as a direct or indirect result of stress. The time required for transported fish to return to normal depends on the degree of stress during transportation. Pacific bluefin tuna (*Thunnus orientalis*) required 72 h to return to their normal status after transportation over 15.2 km [12], and *T. rubripes* required 168 h to physically recover from 30 min of transportation stress [10].

The fish intestine is the main site of the transformation and excretion of xenobiotics, and it plays a crucial role in stress responses and maintaining homeostasis [13]. Previous studies have demonstrated that transport stress induces a series of stress responses in the intestinal tract [14,15]. In rainbow trout (*Oncorhynchus mykiss*), the abundance of the chaperone protein HSC70 in the epithelia of the intestine was generally higher in transport-stressed fish than in control fish [16]. In sea bass (*Lateolabrax maculatus*), 72 h of transport at a density of 250 kg/m^3^ severely affected the antioxidant and immune systems and energy metabolism, and damaged organs including the liver, gills, and gut [17]. The effects of transport stress on the intestinal tract cannot be ignored. However, the responses of the yellow catfish intestine to transport stress remain largely unknown.

The development of high-throughput RNA-seq technology has made it possible to analyze the transcriptional landscape to provide insights into the mechanisms of the response to transportation in fish [18]. Transcriptome analyses of zebrafish (*Danio rerio*) liver revealed that transport stress up-regulates genes related to gluconeogenesis, and down-regulates genes encoding specific immune components, genes related to glycolysis, genes encoding peroxisomal enzymes that produce reactive oxygen species (ROS), and genes encoding xenobiotic-metabolizing enzymes [19]. In addition, the dynamic balance of intestinal flora is important for fish health, as the gut microbiota plays an indispensable role in maintaining nutrient supply to the host, the host’s physiological processes, and stress responses [20]. Various stressors can affect the composition of the intestinal microbial community, consequently affecting fish health [21]. In cobia (*Rachycentron canadum*), transport stress was found to significantly reduce the taxonomic diversity of the gut microbiome, reduce the abundance of healthy microbiota, and increase the abundance of opportunistic pathogens [22].

Hybrid yellow catfish, a member of the family Siluriformes, is an important fresh water fish. This species is economically valuable for aquaculture because it is popular to consumers, so it is widely farmed [5]. However, it is highly susceptible to transport stress. In the current study, we analyzed intestinal biochemical parameters and mucus cells in transported hybrid yellow catfish, and conducted transcriptome analyses of the intestine using high-throughput RNA-seq. The intestinal microbiota was analyzed using 16S rDNA sequencing. The overall aim of the study was to obtain new information about the negative effects of transport stress on hybrid yellow catfish, with a view to improving transportation methods.

## 2. Materials and Methods

### 2.1. Fish and Experimental Design

Hybrid yellow catfish (average body weight 7.06 ± 1.30 g and length 5.06 ± 0.82 cm) were collected from the Yixing Base of Freshwater Fisheries Center, Chinese Academy of Fishery Sciences (Wuxi, China). Before the experiment, the fish were acclimated in a recirculating water system (pH 7.5 ± 0.3; dissolved oxygen > 5.5 mg/L; 26 ± 3 °C; ammonia nitrogen and nitrite < 0.01 mg/L) for 1 week. During this period, the fish were fed with commercial feed (crude fat 6%, crude protein 38.0%) at 2% (*w*/*w*) of their body weight twice per day. Food was withheld for 24 h before the simulated transportation experiment. We used an automated shaker (130 × 110 × 70 cm) (Mince instrument, Changzhou, China) for the simulated transportation treatments. Eighteen transport bags (three per treatment group) were placed on the shaker platform. Each transport bag was 40 × 82 cm and contained 30 fish. Each bag was filled with a one-third volume of water, the fish were added, and then the bag was filled with oxygen. The simulated transportation treatment times were 0 h, 2 h, 4 h, 8 h, and 16 h with shaking at 100 rpm. The recovery group consisted of fish that were allowed to recover for 96 h after 16 h of simulated transportation. The recovery environment was the same as that of the control group.

### 2.2. Sample Collection

At each time point, 12 fish were randomly selected from the three transportation bags per treatment group. At the same time, fish were sampled from the recirculating aquaculture system as the control. The fish were anaesthetized with 200 mg/L MS-222, and the posterior section of the bowel was collected. The samples for analyses of biochemical parameters, the transcriptome, and intestinal microbiota were rapidly frozen in liquid nitrogen and then stored at −80 °C. Samples of fish from the transport groups (0 h, 2 h, 4 h, 8 h, 16 h) and recovery group (96 h) were rapidly placed in Bouin’s solution for histological analyses. Transcriptome and 16S sequencing were conducted using portions of samples from the 0-h group (Ctrl), 16-h transport group (Str), and 96-h recovery group (Rec). Biochemical analyses were conducted using samples from the transportation groups (0 h, 2 h, 4 h, 8 h, 16 h), recovery group (96 h), and corresponding controls.

### 2.3. Intestine Biochemical Analyses

The intestine samples were homogenized with nine volumes of normal saline [23] and then the mixture was centrifuged (3000× *g* for 10 min at 4 °C). The supernatant was used for the analyses. The total antioxidant capacity (T-AOC), malondialdehyde (MDA) content, and activities of superoxide dismutase (SOD) and catalase (CAT) were determined using commercial kits purchased from the Nanjing Jiancheng Bioengineering Institute (Nanjing, China) [24].

### 2.4. Histological Analyses of intestine

The tissue samples were fixed in Bouin’s solution for 24 h [25,26] and then immersed in 70% *v*/*v* ethanol. The samples were then dehydrated with a stepwise ethanol gradient (*v*/*v*: 70% ethanol, 80% ethanol, 95% ethanol, and anhydrous ethanol). For clearing, the samples were immersed in a mixture of xylene and anhydrous ethanol (1:1) for 1 h, then in xylene I for 2 h and xylene II for 2 h. The wax-soaked tissues were embedded using a Leica EG 1150 H embedding machine and then cut into 7-µm sections before being dried at room temperature and heated to 60 °C for 1 h. The sections were dewaxed by successive immersion in xylene I, xylene II, 100% *v*/*v* alcohol, 95% *v*/*v* alcohol, 90% *v*/*v* alcohol, 80% *v*/*v* alcohol, and 70% *v*/*v* alcohol solution, and then stained with Alcian blue-periodic acid-Schiff (AB-PAS). After sealing with neutral gum, the stained sections were observed and photographed under a microscope (E100, Nikon, Tokyo, Japan) [26].

### 2.5. Intestinal Transcriptomic Sequencing and Analysis

#### 2.5.1. RNA Isolation and Transcriptome Sequencing

Intestine samples were collected from three individuals in the Ctrl group, three in the Str group, and three in the Rec group using a TRIzol kit (Invitrogen, Carlsbad, CA, USA). The purity, concentration, and structural integrity of RNA were determined, and then RNA-Seq libraries were constructed. Sequencing was conducted on the Illumina NovaSeq 6000 platform (Illumina Inc., San Diego, CA, USA).

#### 2.5.2. Assembly and Annotation of Transcripts, Identification of Differentially Expressed Genes

Raw data were processed using Cutadapt v1.9 and then clean data were mapped to the reference genome of *Tachysurus fulvidraco* (https://www.ncbi.nlm.nih.gov/genome/?term=yellow+catfish (accessed on 6 October 2020)). The differentially expressed genes (DEGs) among the Ctrl group, Str group, and Rec group were identified using DESeq with the following screening criteria: |log 2 (fold change)| > 1 and *p* < 0.05. The DEGs were subjected to GO (Gene Ontology, http://geneontology.org/ (accessed on 20 November 2021)) and KEGG enrichment analyses (Kyoto Encyclopedia of Genes and Genomes, http://www.kegg.jp/ (accessed on 20 November 2021)). The significantly enriched GO terms and KEGG pathways (adjusted *p* < 0.05) were used to analyze the potential function of the DEGs. The raw data have been deposited in the GEO database at the NCBI (accession number: GSE206148).

#### 2.5.3. qRT-PCR Analyses

To verify the transcriptome sequencing results and further analyze the intestinal response to transport stress, the transcriptional patterns of nine genes in the intestinal tissues of three groups (Ctrl, Str, and Rec) were validated by a real-time quantitative PCR (qRT-PCR). The total RNA was extracted as described in Section 2.5.1. Then, cDNA was synthesized from the extracted RNA using PrimeScript RT Master Mix (Takara, Dalian, China). The qRT-PCR analyses were performed in a total volume of 20.0 μL containing 0.5 μL of each primer, 2 μL of cDNA, 7.0 μL of ddH_2_O, and 10 μL of TB Green Premix Ex Taq Ⅱ (Takara). The thermal cycling conditions for the qRT-PCR were as follows: denaturation at 95 °C for 30 s, then 40 cycles of 95 °C for 5 s, and 60–63 °C for 1 min. The 2^–ΔΔCt^ method was used to calculate relative gene transcript levels. *β*-actin was used as the internal control.

### 2.6. Intestinal Microbiome Sequencing and Analysis

First, DNA was extracted from the intestinal samples of 30 hybrid yellow catfish in three groups (10 each from Ctrl, Str, and Rec) using the E.Z.N.A. ^®^Stool DNA Kit (D4015, Omega Inc., Norcross, GA, USA). The PCR mixture consisted of 25 ng template DNA, 12.5 μL of PCR Premix, 2.5 μL of each primer, and PCR-grade water to complete the volume to 25 μL. The PCR conditions included an initial denaturation at 98 °C for 30 s, followed by 32 cycles of denaturation of 98 °C for 10 s, 54 °C for 30 s, and extension at 72 °C for 45 s, and then a final extension at 72 °C for 10 min. The PCR products were purified using AMPure XT beads (Beckman Coulter Genomics, Danvers, MA, USA) and then used to prepare a sequencing library. The minimum concentration of product for the library construction was 2 nmol/L.

The constructed libraries were sequenced on the NovaSeq PE250 platform. Raw data have been deposited in the SRA database at the NCBI (accession number: PRJNA853144). The raw reads were quality-filtered to obtain clean tags. Chimeric sequences were filtered using Vsearch software (v2.3.4) (accessed on 20 November 2021). The feature table and feature sequences were obtained after dereplication using DADA2. Sequences with higher than 97% similarity were grouped into the same OTU (operational taxonomic unit). Blast was used for sequence alignments. Feature abundance was normalized to the relative abundance in each sample with the SILVA classifier (release 132). Alpha diversity and beta diversity were analyzed by QIIME2, and figures were constructed using R (V3.5.2). Differences among compared groups were determined on the basis of obtained species abundance statistics. Alpha diversity, as represented by five indices (Chao1, observed species, Goods coverage, Shannon’s, and Simpson’s) provided an indication of the complexity of species diversity under transport stress. A constrained principal component analysis (PCoA) of Bray–Curtis distances was used to determine differences in species complexity among multiple groups. A linear discriminant analysis (LDA) effect size (LEfSe) revealed differences in the abundance of bacterial taxa among the Ctrl, Str, and Rec groups. The Kruskal–Wallis test was used to adjust for multiple tests (*p* < 0.05) and for the effect size analysis (LDA score > 3). A PICRUSt2 analysis was performed using tools from OmicStudio https://www.omicstudio.cn/analysis/ (accessed on 20 November 2021). The function of intestinal flora was predicted on the basis of KEGG functional annotations.

### 2.7. Intestinal Microbiota and DEGs Correlation Analyses

Pearson’s correlation analyses were performed to detect relationships between DEGs and intestinal bacteria. The R package (version 3.6.3) was used to correct *P* values for multiple comparisons. Differences at *p* < 0.05 were considered to be statistically significant, and those at *p* < 0.01 were considered to be highly significant. A cluster correlation heatmap was constructed to display correlation information—the darker the color in the figure, the stronger the correlation; warm colors represent positive correlations, and cool colors represent negative correlations. In the figure, the significance of correlation values is indicated by asterisks, with more asterisks denoting stronger significance.

### 2.8. Statistical Analyses

Data were analyzed using SPSS 26.0 (SPSS Inc., Chicago, IL, USA). The results shown in figures and tables are the mean ± standard error of the mean (SEM). Data were tested for normality and homogeneity of variance using Shapiro–Wilk’s and Levene’s median tests, respectively. A one-way analysis of variance (ANOVA) followed by Duncan’s multiple range test were used to detect significant differences in biochemical indexes and gene transcript levels in the same group among different transport times. An independent-samples *t* test was used to compare the treatment group with the control group at each time point. The level of significance was *p* < 0.05. The significance of differences in microbial alpha diversity and abundance among the samples was determined by Wilcoxon’s rank sum test. Correlations between intestinal bacteria and DEGs were analyzed using Spearman’s r correlation analyses, as described in Section 2.7.

## 3. Results

### 3.1. Intestine Biochemical Parameters

**Figure 1:**
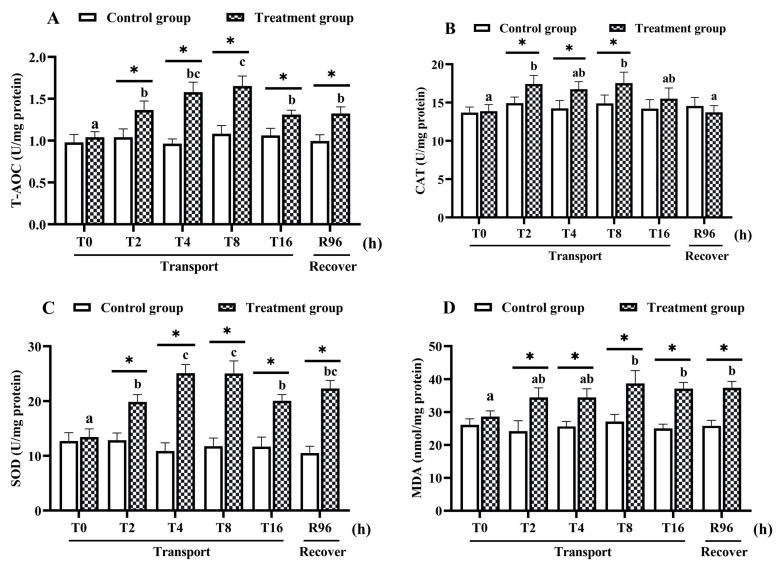
Intestinal antioxidant-related biochemical indices of hybrid yellow catfish under transport stress (*n* = 9 replicates). (**A**) Total antioxidant capacity, T-AOC; (**B**) catalase, CAT; (**C**) superoxide dismutase, SOD; (**D**) malondialdehyde, MDA. *X*-axis indicates the treat time. The values were expressed as mean ± SEM. Asterisks (*) mean statistical significance (*p* < 0.05) between control groups and transportation groups. Different letters indicate statistical significance (*p* < 0.05) among different transport groups.

As shown in Figure 1, the T-AOC, SOD and CAT activities, and MDA content did not differ significantly over time in the control group. However, their levels were higher in the transportation group than in the control group from 2 h of transportation onwards. During the simulated transport treatment, the T-AOC increased to reach its maximum value at 8 h, then significantly decreased at 16 h (Figure 1A; *p* < 0.05). The CAT activity was higher in the transportation group than in the control group at 2 h, 4 h, and 8 h, but decreased to a level similar to that in the control at 16 h of transportation (Figure 1B; *p* < 0.05). The SOD activity also increased to its peak at 8 h of transportation but decreased at 16 h (Figure 1C; *p* < 0.05). The MDA activity was significantly higher in the transportation group than in the control group at 8 h and 16 h (Figure 1D; *p* < 0.05). After recovery for 96 h, the T-AOC, SOD activity, and MDA content were still significantly higher than their respective values in the control group, but not significantly different from their respective values in the 16-h transportation group.

### 3.2. Histological Analyses

**Figure 2:**
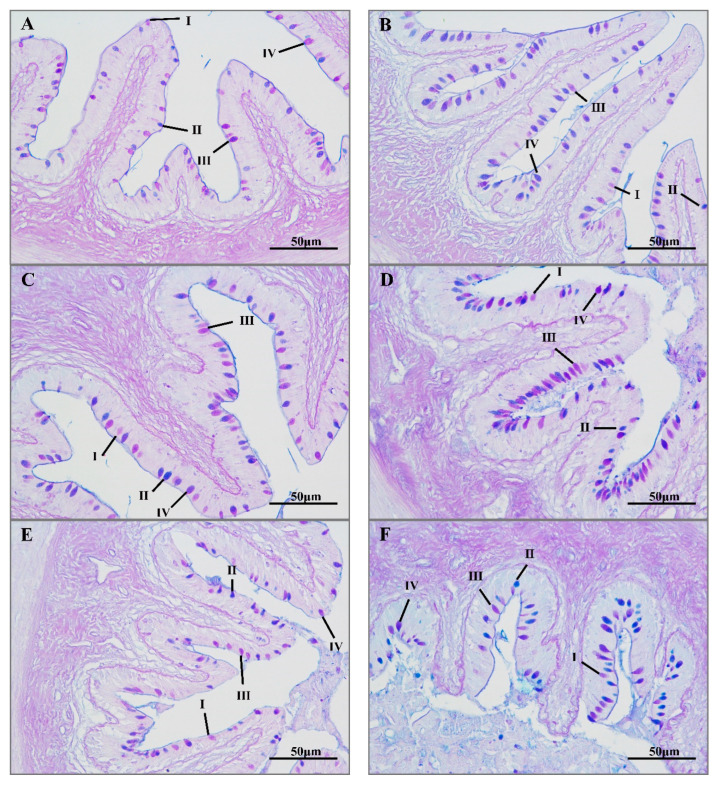
Alcian blue-periodic acid-Schiff staining (AB-PAS) of hybrid yellow catfish intestine under transport stress. Intestine mucous cells at different transport times 0 h (**A**); 2 h (**B**); 4 h (**C**); 8 h (**D**); 16 h (**E**); 96 h recovery group (**F**). Ⅰ, Ⅱ, III and Ⅳ represent different mucous cell types.

In the histological analyses, the AB-PAS staining of intestinal samples revealed type I, II, III, and IV mucus cells. There were significantly more mucus cells in the transportation and recovery groups than in the control group, with the maximum number in the 8-h transportation group. After 8 h of transport, the number of mucus cells decreased significantly. Most of the mucus cells in the control group were type IV and III. With increasing transport time, the number of type II mucus cells gradually increased (Figure 2).

### 3.3. Intestinal Transcriptome Analysis

#### 3.3.1. Transcriptome Sequencing, Assembly, and Statistics

Intestinal transcriptome data have been deposited in the NCBI GenBank (accession number: GSE206148). Nine mRNA libraries (Ctrl-1, Ctrl-2, Ctrl-3, Str-1, Str-2, Str-3, Rec-1, Rec-2, Rec-3) from fish in the Ctrl group, Str group, and Rec group were prepared and sequenced. As shown in Table S1, there were 42,235,166; 39,847,858; 41,218,400; 36,627,760; 48,324,180; 40,156,618; 39,748,556; 39,320,374; and 33,010,852 valid reads in the Ctrl-1, Ctrl-2, Ctrl-3, Str-1, Str-2, Str-3, Rec-1, Rec-2, and Rec-3 libraries, respectively (84.35–95.53% valid reads; Q20 values of 99.91–99.97%; Q30 values of 97.44%–97.76%, and GC contents of 45–46.5%). The numbers of reads mapped to the reference genome were 39,010,100 (Ctrl-1); 36,837,895 (Ctrl-2); 381,148,448 (Ctrl-3); 36,875,748 (Str-1); 36,111,813 (Str-2); 30,627,561 (Str-3); 33,889,373 (Rec-1); 44,897,392 (Rec-2); and 37,383,773 (Rec-3).

#### 3.3.2. Selection of DEGs

**Figure 3:**
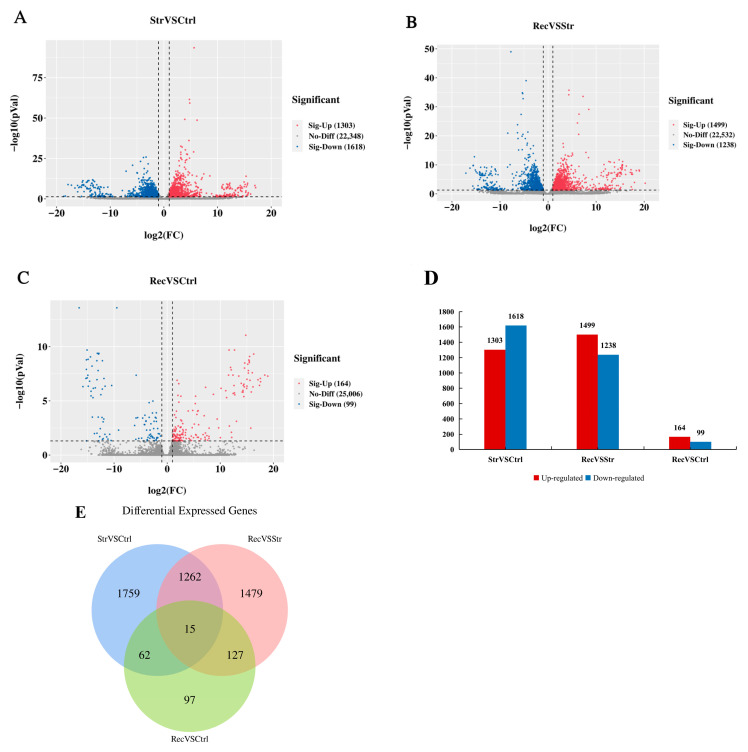
(**A**) Volcano plot of DEGs in StrVSCtrl (transport 16h group vs. control group). (**B**) Volcano plot of DEGs in RecVSStr (recovery group vs. transport 16h group). (**C**) Volcano plot of DEGs in RecVSCtrl (recovery group vs. control group). Gray dots represent genes showing no significant differences in expression. Red and blue dots represent significantly up-regulated and down-regulated DEGs, respectively. (**D**) Transcriptome analysis of the number and expression of DEGs. (**E**) Venn diagram of the number of DEGs.

We identified the DEGs in pairwise comparisons between the Ctrl, Str, and Rec libraries using the screening criteria: |log2(foldchange)| ≥ 1, *p* < 0.05. We identified 3134 DEGs (1383 up-regulated and 1751 down-regulated) between the Str and Ctrl libraries; 2883 DEGs (1579 up-regulated and 1304 down-regulated) between the Rec and Str libraries; and 301 DEGs (183 up-regulated and 118 down-regulated) between the Rec and Ctrl libraries (Figure 3A–D). A Venn diagram shows the number of DEGs in each comparison (Figure 3E).

#### 3.3.3. GO and KEGG Enrichment Analyses of DEGs

**Figure 4:**
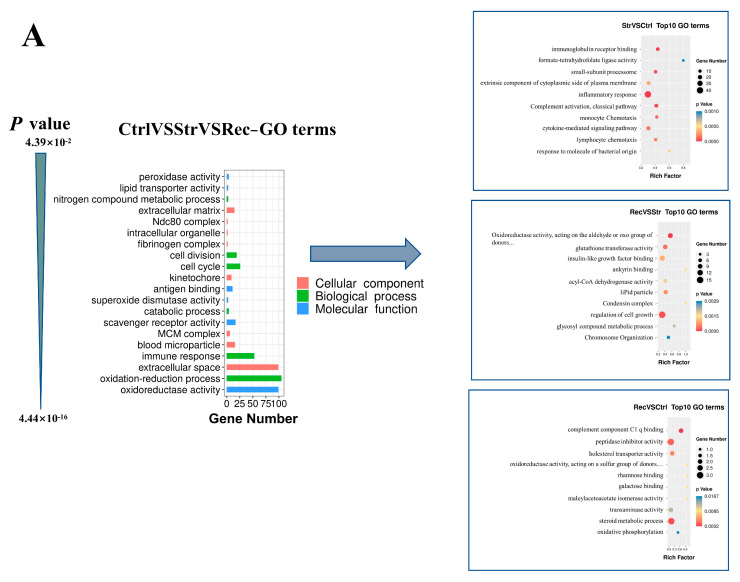

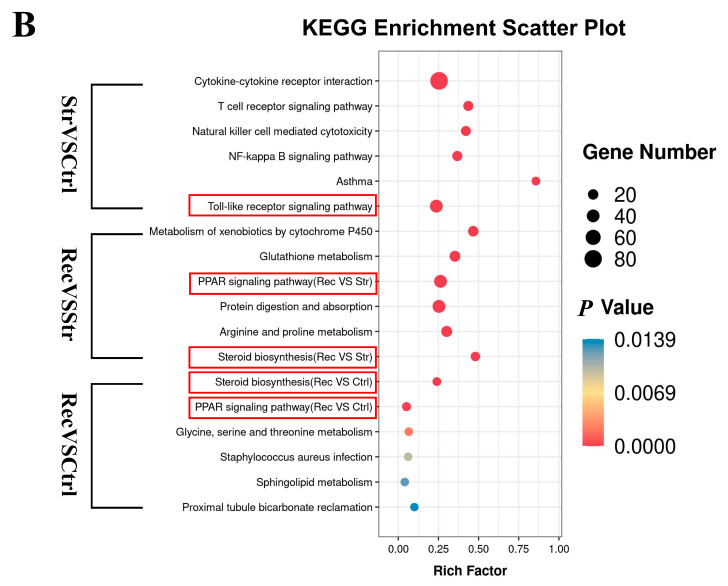
(**A**)The Gene Ontology catalogs with significant enrichment of differential genes were screened, respectively, from MF (molecular function), CC (cellular component), and BP (biological process) of Ctrl (control group) vs. Str (transport 16 h group) vs. Rec (recover group), Str vs. Ctrl, Rec vs. Str, and Rec vs. Ctrl, according to *p* values. (**B**) The top 6 enrichment KEGG pathways with significant enrichment of differential genes were screened, respectively, from Str (transport groups) vs. Ctrl (control groups), Rec (recover groups) vs. Str (transport groups) and Rec vs. Ctrl according to *p* values.

The DEGs were grouped into molecular function (MF), cellular component (CC), and biological process (BP) categories in the GO enrichment analysis (Figure 4A). In the Ctrl vs. Str vs. Rec comparison, the MF subcategories most enriched with DEGs were oxidoreductase activity, chemokine activity, and interleukin-1 receptor binding; the CC subcategories most enriched with DEGs were extracellular space, extracellular region, and the external side of the plasma membrane; and the BP subcategories most enriched with DEGs were oxidation-reduction process, immune response, and metabolic process. In the Str vs. Ctrl comparison, the MF subcategories most enriched with DEGs were chemokine activity, tumor necrosis factor receptor binding, and immunoglobulin receptor binding; those in the CC category were extracellular space, the external side of the plasma membrane, and blood microparticle; and those in the BP category were defense response to bacterium, inflammatory response, and immune response. In the Rec vs. Str comparison, the MF subcategories most enriched with DEGs were oxidoreductase activity, acting on the aldehyde or oxo group of donors, NAD or NADP as acceptor, and aldehyde dehydrogenase (NAD) activity; those in the CC category were extracellular region, chylomicron, and MCM complex; and those in the BP category were metabolic process, oxidation-reduction process, and mitotic chromosome condensation. In the Rec vs. Ctrl comparison, the MF subcategories most enriched with DEGs were complement component C1q binding, virion binding, and peptidase inhibitor activity; those in the CC category were condensed chromosome outer kinetochore, blood microparticle, and intermediate filament; and those in the BP category were cholesterol metabolic process, the positive regulation of nitric oxide biosynthetic process, and the negative regulation of exo-alpha-sialidase activity.

We conducted a KEGG pathway enrichment analysis for the DEGs in the Ctrl vs. Str vs. Rec comparisons. The results revealed that the transport treatments mainly affected the immune and metabolic pathways (Figure 4B). The Toll-like receptor signaling pathway (TLRs), peroxisome proliferator-activated receptor (PPAR) signaling pathway, and steroid biosynthesis pathway were enriched with DEGs in the Ctrl vs. Str vs. Rec comparisons. In addition, 52, 45, and 12 signaling pathways were enriched with DEGs in the Str vs. Ctrl, Rec vs. Str, and Rec vs. Ctrl comparisons, respectively. The pathways most significantly enriched with DEGs in Str vs. Ctrl were immune-related signaling pathways such as the cytokine–cytokine receptor interaction and the TLRs signaling pathway. In Rec vs. Str, the pathways significantly enriched with DEGs were metabolic signaling pathways such as the PPAR signaling pathway and protein digestion and absorption. Fewer pathways were significantly enriched with DEGs in the Rec vs. Ctrl comparison. Those significantly enriched with DEGs were metabolic signaling pathways such as steroid biosynthesis and the PPAR signaling pathway.

#### 3.3.4. Expression Patterns of DEGs Involved in TLRs Signaling, PPAR Signaling, and Steroid Biosynthesis Pathways under Transport Stress

Nine DEGs in the TLRs signaling pathway, PPAR signaling pathway, and steroid biosynthesis pathway were selected for validation of the transcriptome sequencing results by a qRT-PCR. The transcriptome sequencing results are shown in Table S2.

**Figure 5:**
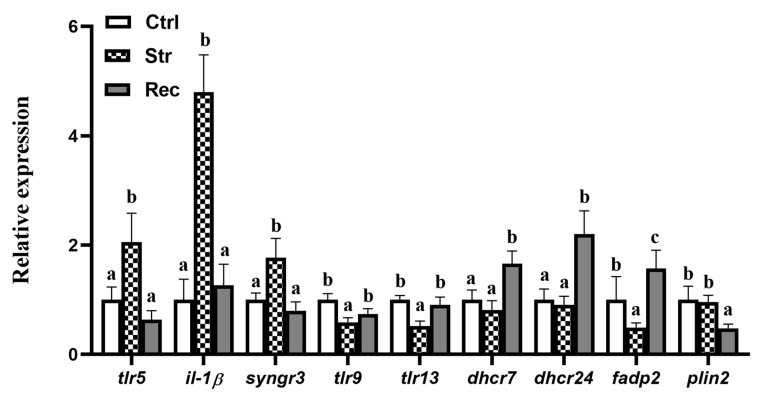
Expression levels of *tlr5*, *tlr9*, *tlr13*, *il1β*, *syngr3*, *fabp2*, *plin2*, *dhcr7* and *dhcr24* mRNA (*n* = 9 replicates) in Ctrl (control groups), Str (transport groups), and Rec (recover groups). Different letters indicate statistical significance (*p* < 0.05) among the three groups.

The five selected DEGs in the TLRs signaling pathway were *tlr5* (encoding toll-like receptor 5); *il-1β* (encoding interleukin 1 beta); *syngr3* (encoding synaptogyrin3); *tlr9* (encoding toll-like receptor 9); and *tlr13* (encoding toll-like receptor 13). The four DEGs in the PPARs signaling pathway and steroid biosynthesis pathway were *dhcr24* (encoding 3β-hydroxysterol-Δ24-reductase); *dhcr7* (encoding 7-dehydrocholesterol reductase); *fabp2* (encoding fatty acid-binding protein, intestinal); and *plin2* (encoding perilipin-2). The primer sequences for these nine genes are listed in Table S3. The results of the qRT-PCR analyses confirmed that, compared with the Ctrl group, the Str group showed increased transcript levels of *tlr5*, *il-1β*, and *syngr3*, and decreased transcript levels of *tlr9* and *tlr13*. Compared with the Str group, the Rec group showed decreased transcript levels of *tlr5*, *il-1β*, and *syngr3*, and increased transcript levels of *tlr9* and *tlr13*. The transcript levels of *dhcr24*, *dhcr7*, and *plin2* were not significantly different between the Str and Ctrl groups. The transcript levels of *dhcr24* and *dhcr7* were significantly higher, and that of *plin2* was significantly lower in the Rec group than in the Str and Ctrl groups. The *fabp2* transcript level was significantly lower in the Str group than in the Ctrl group and was significantly higher in the Rec group than in the Str and Ctrl groups (Figure 5).

### 3.4. Intestinal Microbial Composition

**Figure 6:**
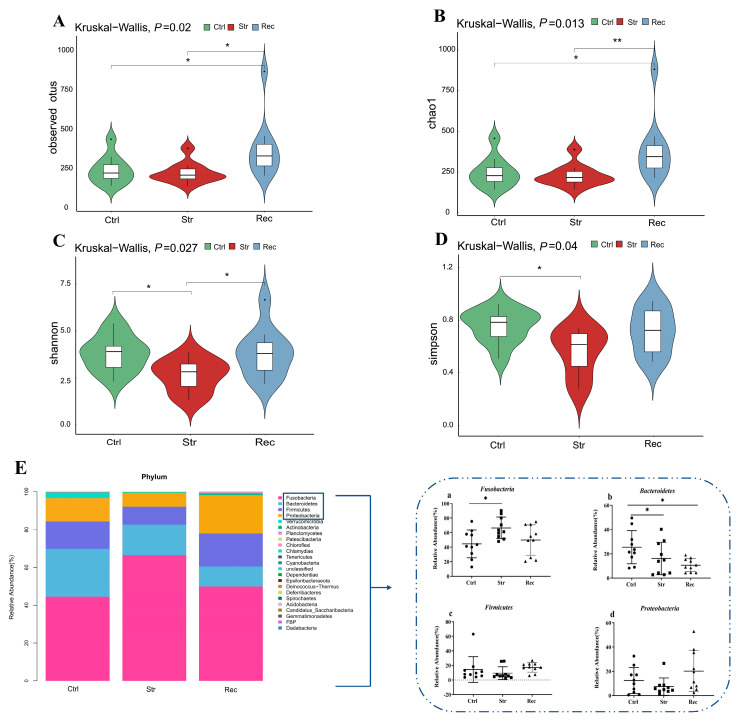
(**A**) Observed species index; (**B**) Chao1 index; (**C**) Shannon’s index; (**D**) Simpson’s index; (**E**) relative abundance of predominant taxa identified at phylum level in control group (Ctrl), transport 16 h group (Str), and recovery group (Rec). Each bar represents relative abundance in each group and 20 most abundant taxa are shown. Relative abundance of four dominate bacterial phyla in three groups showed in (**a**–**d**). Each shape represents relative abundance in each sample. * (*p* < 0.05) and ** (*p* < 0.01) indicate significant difference in abundance.

As shown in Figure S1, the ends of the rarefaction curves were flattened, so we concluded that the sequencing data were suitable for further analyses. In terms of alpha diversity, the observed species index (Figure 6A) and Chao1 index (Figure 6B) were slightly lower in the Str group than in the Ctrl group. Shannon’s index (Figure 6C) and Simpson’s index (Figure 6D) were significantly lower in the Str group than in the Ctrl group. For the Rec group, Shannon’s index, and Simpson’s index were similar to their respective values in the Ctrl group, and the observed species index and Chao1 index were significantly higher than in the Ctrl group.

To explore the effects of transport stress on beta diversity, we conducted a PCoA to explore the relationships among samples based on intestinal microbial community structure. The bacterial community showed clear differences at the phylum and genus levels among the Ctrl, Str, and Rec groups. The dominant phyla in the intestinal samples of these three groups were Fusobacteria, Firmicutes, Bacteroidetes, and Proteobacteria (Figure 6E). Compared with the Ctrl group, the Str group showed an increased relative abundance of Fusobacteria and a decreased relative abundance of Bacteroides (Figure 6E(a,b). The relative abundance of Firmicutes and Proteobacteria was not significantly different among the three groups (Figure 6E(c,d). The ratio of Bacteroides/Firmicutes was significantly lower in the Str and Rec groups than in the Ctrl group.

**Figure 7:**
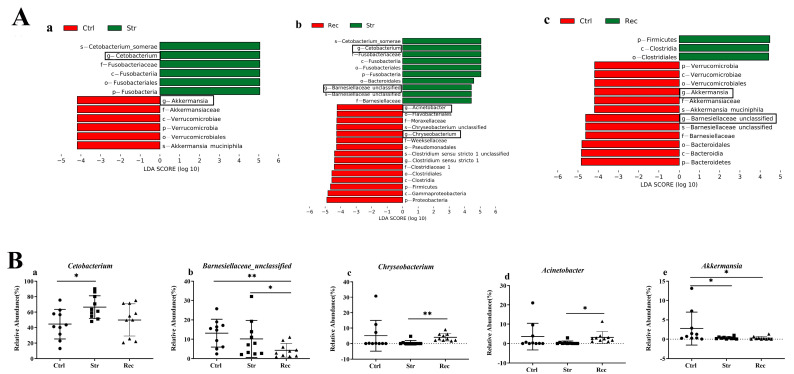
(**A**) Taxa shown in histogram were determined to differ significantly in abundance among different groups by Kruskal-Wallis test (*p* < 0.05) and have LDA score > 4 ((**a**): Ctrl vs. Str; (**b**): Rec vs. Str; (**c**): Ctrl vs. Rec). (**B**) Abundance of dominate bacterial genera (**a**–**e**) in Ctrl, Str, and Rec (*, *p* < 0.05; **, *p* < 0.01).

The abundance of bacterial taxa was compared among the Ctrl, Str, and Rec groups by a LEfSe analysis (Figure S2). At the genus level (Figure 7A), bacteria with high relative abundance such as *Cetobacterium*, *Barnesiellaceae*, *Chryseobacterium*, *Acinetobacter*, and *Akkermansia* differed among the three groups (Figure 7B). The abundance of *Cetobacterium* was higher in the Str group than in the Ctrl group, but similar between the Rec and Ctrl groups (Figure 7B(a)). The abundance of *Barnesiellaceae* was slightly lower in the Str group than in the Ctrl group, and markedly lower in the Rec group than in the Ctrl group (Figure 7B(b)). The abundance of *Chryseobacterium* and Acinetobacter was not significantly different between the Str and Ctrl groups but was significantly higher in the Rec group than in the Str group (Figure 7B(c,d)). *Akkermansia* showed significantly decreased abundance in the Str and Rec groups compared with the Ctrl group (Figure 7B(e)).

**Figure 8:**
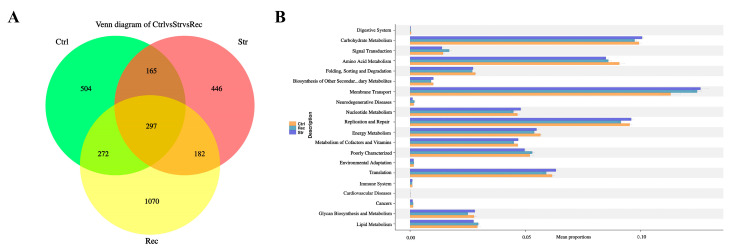
(**A**) Venn diagram analysis depicting the numbers of shared and unique OTUs in control groups, transport groups, and recovery group. (**B**) The abundance ratio of gut microbiota among the control groups, transport groups, and recovery group predicted the level 2 function.

As shown in Figure 8A, the Str group had the fewest OTUs and the Rec group had the largest number of exclusive functional OTUs. At KEGG level 2 (Figure 8B), the most enriched categories of intestinal microbial function in the Ctrl, Str, and Rec groups were “membrane transport”, “carbohydrate metabolism”, “amino acid metabolism”, “replication and repair”, and “translation”.

### 3.5. Relationships between Intestinal Microbiota and DEGs under Transport Stress

**Figure 9:**
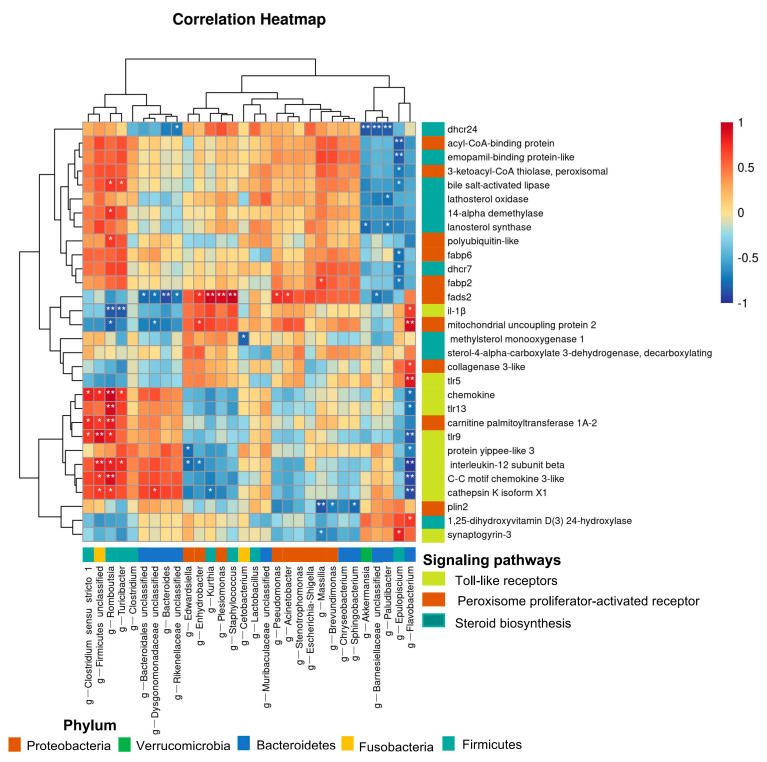
Significant correlation between intestinal bacteria at genus level and differentially expressed genes of toll-like receptors, peroxisome proliferator-activated receptor, and steroid biosynthesis signaling pathways (*, *p* < 0.05; **, *p* < 0.01).

We conducted correlation analyses to detect relationships between intestinal microbes at the genus level and DEGs related to immunity and metabolism. We found that *Firmicutes-unclassified* and *Rombouisia* were positively correlated with cathepsin K isoform X1, C-C motif chemokine 3-like, interleukin-12 subunit beta, *tlr9*, and carnitine palmitoyltransferase 1A-2. *Rombouisia* was positively correlated with *tlr13*, mitochondrial uncoupling protein 2, *il-1β*, polyubiquitin-like, 14-alpha demethylase, and bile salt-activated lipase. *Flavobacterium* was positively correlated with 1,25-dihydroxyvitamin D (3) 24-hydroxylase, *tlr5*, collagenase 3-like, mitochondrial uncoupling protein 2, and *il-1β*; and negatively correlated with chemokine, *tlr13*, *tlr9*, protein yippee-like 3, interleukin-12 subunit beta, C-C motif chemokine 3-like, and cathepsin K isoform X1. *Epulopiscium* was positively correlated with synaptogyrin-3 and negatively correlated with *fabp2*, *dhcr7*, *fabp6*, bile salt-activated lipase, 3-ketoacyl-CoA thiolase, peroxisome, emopamil-binding protein-like, and acyl-CoA-binding protein. The *fads2* gene was negatively correlated with *Bacteroidetes*-unclassified, *Dysgonomonadaceae*-unclassified, *Bacteroides*, *Rikenellaceae*-unclassified, and *Barnesiellaceae*-unclassified; and positively correlated with *Enhydrobacter*, *Kurthia*, *Plesiomonas*, *Staphylococcus*, *Pseudomonas*, and *Acinetobacter*. The gene encoding interleukin-12 subunit beta was negatively correlated with *Edwardsiella*, *Enhydrobacter*, and *Flavobacterium* and positively correlated with *Firmicutes*-unclassified, *Rombouisia*, and *Turicibacter*. The gene-encoding chemokine was negatively correlated with *Flavobacterium* and positively correlated with *Clostridium-sensu-stricto*, *Firmicutes*-unclassified, *Rombouisia*, and *Turcibacter* (Figure 9).

## 4. Discussion

Transport stress can disrupt the normal physiological functions of fish, and this can cause huge economic losses [5]. In this study, the effects of transport stress on the antioxidant, immune, and metabolic function of hybrid yellow catfish were investigated through comprehensive analyses of the hybrid yellow catfish intestine and its microbiota.

### 4.1. Changes in the Intestinal Antioxidant System under Transport Stress

Many studies have reported that transportation stress leads to oxidative stress in fish [27], with an overproduction of ROS and changes to components of the antioxidant system [28]. The T-AOC and the activities of CAT and SOD are indicators of the function of the antioxidant defense system. In addition, MDA, which is a product of lipid peroxidation, is an indicator of the degree of oxidative stress imposed by exogenous stressors [24]. In common carp, 6 h of transport led to oxidative stress, as indicated by enhanced plasma SOD and CAT activities and increased MDA levels [29]. In juvenile rainbow trout, 2 h of transport resulted in enhanced MDA levels and T-AOC in the gills [30]. In our previous study, we detected an increase and then a decrease in SOD activity and T-AOC in the gills of yellow catfish during a 16-h transport treatment [6]. In the present study, we found that the T-AOC, CAT and SOD activities, and the MDA level in the intestine significantly increased with increasing transportation time, but T-AOC and SOD activity significantly decreased by 16 h of transport. These findings suggest that transportation led to oxidative stress, followed by the consumption of antioxidant substances and/or impairment of the antioxidant system, ultimately resulting in decreased antioxidant capacity at 16 h of transport, similar to the findings of our previous study [5]. The oxidative stress induced by transportation may result in mitochondrial dysfunction, because ROS are mainly formed by the mitochondrial electron transport chain in cells [31]. The observed changes in antioxidant enzyme activities and MDA levels revealed that fish experienced oxidative stress during transport, and that the antioxidant system, although activated, failed to fully neutralize free radicals and other pro-oxidants with prolonged transport times [32].

### 4.2. Changes in Mucus Cells in the Intestine under Transport Stress

The mucosal barrier of the intestine is an important line of defense. The mucus cells produce and secrete mucins that lubricate and protect intestinal epithelial cells from damage [33]. It is well established that mucus cells secrete more mucus under stress. Fish are affected by many stressors during transportation, such as increases in total ammonia–nitrogen and nitrite–nitrogen concentrations in the water with prolonged transport times [5]. When treated with un-ionized ammonia (25.81 mg/L), the number of mucus cells in zebrafish (*Danio rerio*) gills first increased and then decreased over the experimental period (24 h, 48 h, and 72 h) [34]. Similarly, there were more mucous cells in the intestine of *Chelon ramada* infected with *Neoechinorhynchus agilis* than in the intestine of uninfected conspecifics [33]. In this study, we found that the number of mucus cells first increased and then decreased during transportation, and the number of type II mucus cells increased gradually with prolonged transport time. In our previous study, we detected a similar trend in the number of skin mucus cells under transport stress [5]. Intestinal microorganisms may indirectly affect the mucus layer by activating host immune cells, or directly affect mucus cell dynamics and the mucus layer by locally releasing bioactive factors [35]. The observed changes in the number of mucus cells and activities of antioxidant enzymes in this study may have resulted from changes in intestinal microbiota that occurred under transport stress. Further studies using mucinous cell and goblet cell models would be useful to clarify the function of different types of mucinous cells and their changes under stress conditions [36].

### 4.3. Transcriptome Analyses Reveal That Transport Stress Affects Intestinal Immune and Metabolic Function

Disturbances in the redox state of cells can activate the immune system. The TLRs are evolutionarily conserved pattern recognition receptors that are involved in altering the innate immune system under stress, and are activated by the damage-associated molecular pattern molecules released under oxidative stress [37,38,39]. Among them, TLR5, TLR9, and TLR13 are important immune sensors. Analyses of gene transcription profiles indicated that *TLR5* was the only TLR gene involved in activating the downstream immune response during transportation. Il-1β is a key proinflammatory cytokine in the downstream immune response. A previous study showed that elevated levels of *il-1β* can greatly enhance the intensity of the inflammatory response [40]. We found that the transcript levels of *tlr5* and *il-1β* were higher in the Str group than in the Ctrl group, suggesting that transportation may enhance the innate immune response through activating *tlr5*, and then an inflammatory response may be initiated by increased transcript levels of *il-1β* in the TLRs signaling pathway. It has been reported that TLR5 plays an integral role in regulating the homeostasis of the intestinal microbiota. The significant increase in TLR5 under transportation stress may have affected the intestinal microbe community. Despite the importance of *TLR5* in host defenses, the extensive activation of *TLRs* may contribute to inflammatory and autoimmune diseases [41]. Therefore, the decreased transcript levels of *tlr9* and *tlr13* may be a protection measure to enhance the mucosal barrier and attenuate intestinal injury and inflammatory responses under transport stress. The inconsistent patterns of *tlr5*, *tlr9*, and *tlr13* transcript accumulation under transport stress were considered to reflect the effectiveness of the innate immune response, with its intricate balance between activation and regulation [42]. In another study, the down-regulation of *syngr3*, which encodes a synaptic vesicle protein, was found to alleviate synaptic dysfunction in mouse and fruit fly primary neurons [43]. In this study, we found that the transcript level of *syngr3* was higher in the Str group than in the Ctrl group—this might be related to synaptic dysfunction caused by transportation stress [44]. In the Rec group, the immune function of hybrid yellow catfish was at least partly restored during the recovery period, as indicated by the lower transcript levels of *tlr5*, *il-1β,* and *syngr3* in the Rec group than in the Str group.

Lipid metabolism is involved in metabolic adaptation under oxidative stress and in the maintenance of the structure and function of biological membranes [45]. Steroid biosynthesis and the PPAR signaling pathway are key pathways related to lipid metabolism and storage [46]. In steroid biosynthesis, *DHCR24* encodes the cholesterol biosynthesis enzyme 3 β-hydroxysteroid-∆ 24 reductase [47], and *DHCR7* encodes a 7-dehydrocholesterol reductase that catalyzes the last step of cholesterol synthesis, i.e., the conversion of 7-dehydrocholesterol into cholesterol. Studies have shown that a high expression of *DHCR24* is related to resistance to apoptosis triggered by oxidative stress, and this gene was found to be up-regulated during the acute response to oxidative stress [48]. In this study, the high transcript levels of *dhcr24* under transport stress may be indicative of increasing resistance to oxidative stress in a cholesterol-dependent manner [49]. At the same time, the expression of DHCR24 could cause cell proliferation, adhesion, and migration [50]. This may be one of the reasons why the number of mucus cells increased in yellow catfish under transport stress. DHCR7, which is regulated by differential alternative splicing and tissue-specific transcription, appears to be unique among enzymes in the post-lanosterol pathway in cholesterol biosynthesis [51]. A previous report revealed that sterol depletion in vivo increased *DHCR7* transcript levels and DHCR7 enzyme activity in rat liver [52]. In this study, the increased transcript level of *dhcr7* under transport stress may have resulted from sterol depletion in vivo, consistent with our previous findings that total blood cholesterol levels were lower in a 16-h transport group than in a control group [6].

PPARs regulate fatty acid biosynthesis and metabolism at the transcriptomic level through the PPAR signaling pathway. In the PPAR signaling pathway, FABP2 mediates fat absorption by the binding and intracellular trafficking of long-chain free fatty acids [53], and PLIN2 is a lipid droplet-associated protein that facilitates cholesterol storage and prevents efflux in macrophages [54]. Other studies detected a relationship between high FABP2 expression and intestinal cell damage [55], and showed that the silencing of intestinal FABP2 decreased toxic lipid accumulation, the inflammatory response, and vascular fibrosis [56]. Moreover, mechanistic experiments revealed that PLIN2 down-regulation led to enhanced autophagic flux and ameliorated ER stress [57]. In this study, the up-regulation of *fabp2* and down-regulation of *plin2* may reflect an anti-inflammatory response to protect against intestinal injury through regulating fatty acid biosynthesis and metabolism under transport stress. In this study, the DEGs in the Rec vs. Str and Rec vs. Ctrl comparisons were mainly related to metabolism. These results indicate that transport stress had a delayed effect on intestinal metabolism.

### 4.4. 16S rDNA Sequence Analyses Reveal Changes in Intestinal Microecology under Transport Stress

Many different types of bacteria work together in the intestinal tract to maintain microecological balance. In this way, the microbial community participates in the regulation of antioxidant, immunity, energy conversion, and other functions in fish [58]. In this study, we found that transport stress affected microbial diversity in the intestine. Two indexes of alpha diversity, Shannon’s index and Simpson’s index, were significantly lower in the Str group than in the Ctrl group, which may have resulted in the instability of the intestinal flora and reduced the disease resistance of hybrid yellow catfish [59].

In this study, the dominant phyla in the intestine were Fusobacteria, Firmicutes, Bacteroidetes, and Proteobacteria. Consistent with these findings, another study detected Fusobacteria, Firmicutes, Bacteroidetes, and Proteobacteria as the dominant phyla in the intestinal tract of hybrid yellow catfish [23]. As dominant phyla in intestinal samples, Fusobacteria and Bacteroidetes showed significant changes in abundance under transport stress. Fusobacteria are anaerobic Gram-negative bacteria whose members are linked with immune suppression, and are involved in inflammation pathways via the recruitment of inflammatory cytokines and the increased production of ROS [60]. We detected a significant increase in the abundance of Fusobacteria in the Str group. This may be related to changes in immunity, inflammation, and antioxidant systems under transport stress. Consistent with our results, other studies have reported that Fusobacteria are associated with host lipid metabolism [61]. Bacteroidetes also are Gram-negative anaerobic bacteria [62] that are closely associated with the occurrence and development of intestinal inflammatory diseases [63]. In this study, transport stress resulted in a reduced abundance of Bacteroidetes and an increase in the Firmicutes/Bacteroidetes ratio in hybrid yellow catfish. The interaction between Bacteroidetes and Firmicutes plays an essential role in energy metabolism in fish intestine [64]. Bacteroidetes harbor carbohydrate-related enzymes that hydrolyze glycoconjugates [65], whereas Firmicutes contribute to the absorption and metabolism of fatty acids in the intestine [66]. Therefore, any change in the Firmicutes/Bacteroidetes ratio induced by transport stress might disrupt the energy balance of hybrid yellow catfish.

*Cetobacterium* (Fusobacteria) and *Barnesiellaceae* (Bacteroidetes) are the dominant intestinal bacterial genera in hybrid yellow catfish [67]. *Cetobacterium* is the most abundant genus in the intestinal microbiota of most fish, and its members are involved in host metabolism and inflammation [68]. Previous studies have shown that exogenous stimuli can induce dysbiosis of the gut microbiota, and aggravate host inflammatory reactions through lipopolysaccharide-related NOD-like receptors and TLRs pathways in fish [69]. *Barnesiellaceae* are related to fat and energy metabolism [70]. After intestinal injury in rats, the beta diversity of intestinal microbes changed, and the relative abundance of *Barnesiellaceae* and *Bacteroidaceae* decreased [71]. The increased abundance of *Cetobacterium* and the decreased abundance of *Barnesiellaceae* observed in this study suggest that transport stress disturbed normal metabolism, leading to intestinal injury in hybrid yellow catfish. In the Rec group, the abundance of Fusobacteria returned to a level similar to that in the Ctrl group, whereas Bacteroidetes were still less abundant than in the Ctrl group. This may be one of the reasons for delayed disease outbreaks after transportation. *Chryseobacterium* and *Acinetobacter* are potential pathogenic bacteria that can threaten fish health [72,73,74]. Among various bacterial diseases, those caused by *Acinetobacter spp*. and *Chryseobacterium spp*. have been recently reported in different fish species [73,75]. In this study, we found that the relative abundance of *Chryseobacterium* and *Acinetobacter* was markedly higher in the Rec group than in the Str group. This may indicate that the risk of disease is increased after transportation. *Akkermansia* is considered to be a promising probiotic because of its ability to ameliorate host immune responses and metabolic function [76]. In this study, transport stress led to a decrease in the relative abundance of *Akkermansia*, suggesting that the functional barrier of the intestine might be disrupted. In summary, these results indicate that transport stress can cause intestinal injury and adversely affect the antioxidant, immune, and metabolism functions of hybrid yellow catfish by altering the intestinal microbial community.

### 4.5. Intestinal Microbiota and Its Relationship with DEGs under Transport Stress

We detected close relationships between some components of the microbiome and metabolism-related and immune-related DEGs. As a group of giant bacteria, *Epulopiscium* can digest certain macronutrients in the host’s gut [77]. For example, in the intestinal tract of tropical herbivorous surgeonfish, *Epulopiscium* play a key role in digestion and pH homeostasis [78]. In the present study, we detected a negative relationship between *Epulopiscium* and metabolism-related genes such as *fabp6*, *dhcr7*, and *fabp2* in the intestine of yellow catfish under transport stress. *Flavobacterium* can cause an immune response and diseases in fish. In the head kidney of Mandarin fish, *Flavobacterium columnare* infection led to an up-regulation of genes encoding CC chemokine 3, interleukin-8, interleukin-1β, and C-X-C motif chemokine 14-like [79]. Similarly, we found that *Flavobacterium* was negatively correlated with *cxcl3*, *il-12*, *chemokine*, *tlr9*, and *tlr13*, and positively correlated with *tlr5* and *il-1β* under transport stress. In addition, *Firmicutes*-unclassified and *Rombouisia* also showed clear relationships with immune-related genes encoding components of the TLRs signaling pathway. Notably, *fads2*, *il-12,* and *chemokine* showed clear correlations with multiple bacterial genera, which highlights the multifaceted pathways of gene–transcriptome interactions. Genome-wide mbQTL studies have shown that the innate host immune system is a major determinant in shaping the microbiome, especially via the innate molecules of pattern recognition receptors, including TLRs. In addition to immune-related genes, those related to the mucus barrier and sugar digestion also affect the gut microbiome. Genetic variations among hosts could lead to intestinal dysbiosis and even disease. Together, these findings suggest that there is a significant interaction between the gut transcriptome and microbiome, which may be mediated by key transcription factors and TLRs, the steroid biosynthesis pathway, and the PPAR signaling pathway under transport stress.

## 5. Conclusions

The results of this study show that transport stress leads to oxidative stress and the dysregulation of lipid metabolism and immunity in hybrid yellow catfish. As all of these processes are interconnected, these changes result in a vicious cycle. Transport stress leads to oxidative stress and compensatory disorders of the innate immune and metabolic functions, with follow-on effects on TLRs signaling, steroid biosynthesis, and the PPAR signaling pathway in the intestinal tract. These alterations affect the composition of the intestinal microbial community. Our findings indicate that interactions between the host (hybrid yellow catfish) and its intestinal microbes are involved in resistance to transport stress. This study provides a theoretical basis for the in-depth exploration of the response mechanism of teleost fish under transport stress.

## Data Availability

The transcriptome sequence datasets [GENERATED/ANALYZED] have been deposited at the NCBI under the accession number GSE206148 [https://www.ncbi.nlm.nih.gov/search/all/?term=GSE206148], and the 16S rRNA datasets are under the accession number PRJNA853144. The raw data supporting the conclusions of this article will be made available by the authors, without undue reservation. Requests to access these datasets should be directed to qiangj@ffrc.cn.

## Supplementary Materials

The following supporting information can be downloaded at: https://www.mdpi.com/article/10.3390/antiox11091737/s1, Figure S1: Rarefaction curves and estimators of the different samples in control group (Ctrl), transport 16h group (Str) and recover group (Rec); Figure S2: Linear discriminant analysis effect size (LEfSe) analysis comparing abundance of all detected bacterial taxa among yellow catfish in control group, transport group and recover group. Table S1: Overview of reads for mRNA-seq and quality filtering; Table S2: Differentially expressed mRNA verified by mRNA-Seq; Table S3: The specific primer sequences for qPCR in this study
