# Supplementary material for: Integrated Transcriptome and 16S rDNA Analyses Reveal That Transport Stress Induces Oxidative Stress and Immune and Metabolic Disorders in the Intestine of Hybrid Yellow Catfish (Tachysurus fulvidraco♀ × Pseudobagrus vachellii♂)
Source: Antioxidants (Basel). 2022 Aug 31;11(9):1737. doi: 10.3390/antiox11091737 (PMC9496016; doi:10.3390/antiox11091737)
Supplement: Supplementary file 1 [file antioxidants-11-01737-s001.zip › antioxidants-1858613-supplementary.pdf]

Table S1. Overview of reads for mRNA-seq and quality filtering

Sample	Raw Data		Valid Data		Valid Ratio	Q20%	Q30%	GC %
	Read	Base	Read	Base				
Ctrl-1	44726962	6.71G	42235166	6.34G	94.43	99.92	97.47	45
Ctrl-2	41713136	6.26G	39847858	5.98G	95.53	99.94	97.55	46
Ctrl-3	43536414	6.53G	41218400	6.18G	94.68	99.92	97.52	46
Str-1	41473770	6.22G	36627760	5.49G	88.32	99.97	97.70	45
Str-2	52389816	7.86G	48324180	7.25G	92.24	99.91	97.47	45.50
Str-3	42216026	6.33G	40156618	6.02G	95.12	99.94	97.76	46.50
Rec-1	42771760	6.42G	39748556	5.96G	92.93	99.95	97.49	46
Rec-2	41857526	6.28G	39320374	5.90G	93.94	99.92	97.44	46
Rec-3	39134716	5.87G	33010852	4.95G	84.35	99.96	97.46	46

Table S2. Differentially expressed mRNA verified by mRNA-Seq. Fold change is equal to the ratio of transport 16 group (Str) mean to control group (Ctrl) mean, recover group (Rec) mean to Str mean and Rec mean to Ctrl mean, where "mean" is the mean of three biological replicates.

**Gene abbreviation**	**Gene description**	**Log2 (flod-change) / Regulation StrVSCtrl**	**Log2 (flod-change) / Regulation RecVSStr**	**Log2 (flod-change) / Regulation RecVSCtrl**
tlr5	toll like receptor 5	4.52/up	-3.79/down	no significant difference
il-1β	interleukin 1 beta	13.97/up	-13.97/down	no significant difference
syngr3	synaptogyrin3	1.21/up	-2.28/down	no significant difference
tlr9	toll like receptor 9	-4.51/down	3.51/up	no significant difference
tlr13	toll like receptor 13	-2.71 /down	2.11/up	no significant difference
dhcr7	7-dehydrocholesterol reductase	no significant difference	2.52/up	1.71/up
dhcr24	delta (24)-sterol reductase	no significant difference	2.52/up	2.87/up
fabp2	fatty acid-binding protein, intestinal	-1.94/down	3.60/up	1.66/up
plin2	perilipin-2	no significant difference	-2.25/down	-1.79/down

Table S3. The specific primer sequences for qPCR in this study

Gene	Primer sequence (5’-3’)	Efficiency%	Amplicon size	GenBank number
tlr5	F: AGCTGGACAGGAGAACCGAT<br>R: TTAGGAGGCAAAACCGGGAC	98.000	182	XM_027135423.1
il-1β	F: GTGCTGAAGGAAAAGACTCCCA<br>R: GCAGTTTGGTGGGTTGTAGG	106.275	105	XM_027139701.1
syngr3	F: CGTGCTACTGGAGATTGGCT<br>R: CCAGTGGCAGTTCGTTAGGT	108.846	110	XM_027142826.1
tlr9	F: GGGCAGGACACAAGGGTTAT<br>R: CCTCCAGACAAAGCCGGAAT	99.639	171	XM_027167061.1
tlr13	F: AGCTTCCGCTACAATCGCAT<br>R: CAGTGAGTGCTTTGCGATCC	101.149	121	XM_027147837.1
dhcr7	F: TGCACCAACATTCTCGGCTA<br>R: TCCGATGCGTGGATTGAACT	107.769	144	XM_017478833.2
dhcr24	F: GCTTTACGAGCAGCATCACG<br>R: AACGGACACAGCCATAAGGG	108.050	117	XM_027179593.1
fabp2	F: AGAAAGCTGGCTGAACACGA<br>R: CGAGCGTGAACTCGATGTCT	96.018	112	XM_017461533.2
plin2	F: GCCTGCCACAAAACATCCAA<br>R: GGACAGGTCGCTTAGAGCTG	103.407	101	XM_027169412.1
β-actin	F: GGATTCGCTGGAGATGATG<br>R: TCGTTGTAGAAGGTGTGATG	99.268	221	XM_027148463.1

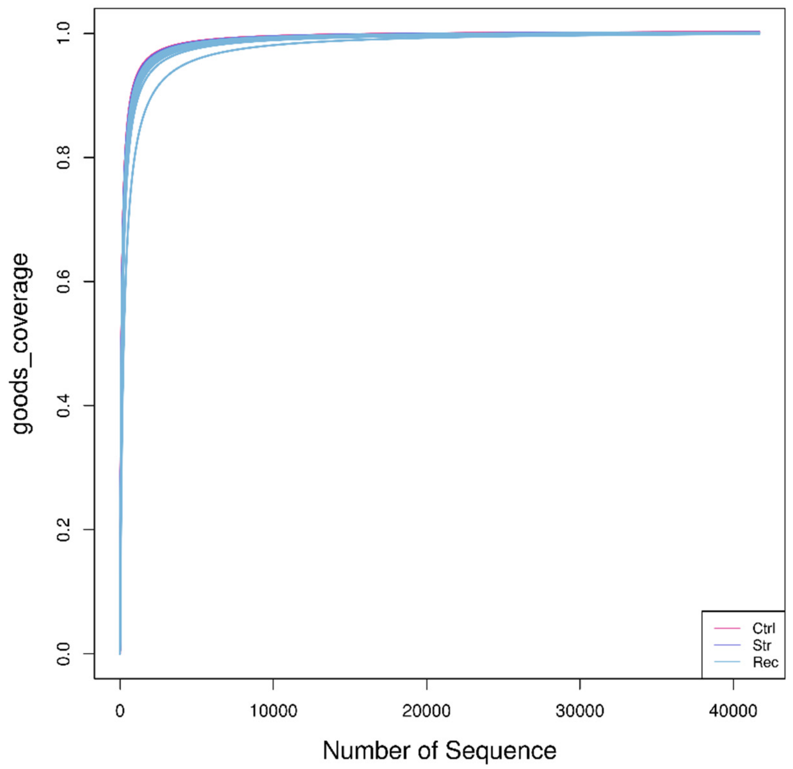

Figure S1: Rarefaction curves and estimators of the different samples in control group (Ctrl), transport 16h group (Str) and recover group (Rec).

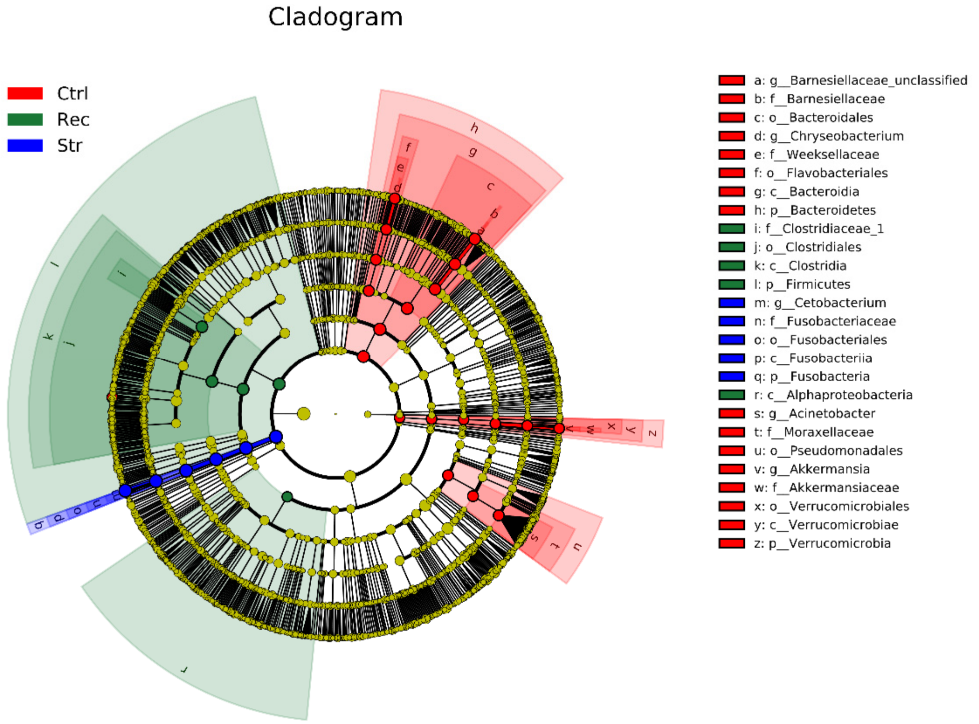

Figure S2: Linear discriminant analysis effect size (LEfSe) analysis comparing abundance of all detected bacterial taxa among yellow catfish in control group, transport group and recover group. Red, blue and green indicate taxa enriched control group (Ctrl), transport 16h group (Str) and recover group (Rec), respectively. Brightness is proportional to abundance of each taxon.
